# Surgeon task load, ergonomics, and clinical outcomes in laparoscopic versus robotic-assisted inguinal hernia repair: a systematic review and meta-analysis of randomized controlled trials

**DOI:** 10.1007/s11701-026-03805-8

**Published:** 2026-10-07

**Authors:** Anna Rümmele-Vogt, Julian Süsstrunk, Marie-Thérèse Böttcher, Johannes Baur, Beat P Müller, Daniel C Steinemann, Fiorenzo V Angehrn

**Affiliations:** 1 Medical Faculty, University of Basel, Basel, Switzerland; 2 Clarunis, Department of Visceral Surgery, Clarunis, University Digestive Health Care Centre, St. Clara Hospital and University Hospital Basel, Basel, Switzerland

**Keywords:** Inguinal hernia repair, Robotic surgery, Hernia, Minimally invasive surgery, Ergonomics, Surgeon workload

## Abstract

Conventional laparoscopic inguinal hernia repair (LIHR) is common and robotic-assisted techniques (RIHR) are increasingly being implemented. Current literature is indecisive on whether RIHR offers clinical and ergonomical benefits. This systematic review compares LIHR and RIHR specifically focusing on surgeon workload and ergonomics. A literature search was performed in four databases to find randomized controlled trials comparing RIHR and LIHR. A risk of bias assessment was conducted using the Cochrane Tool and results were synthesized as pooled estimate, odds ratio or narrative review. Four trials were identified, encompassing 482 patients. The groups showed similar baseline characteristics across studies. A broad range of surgeon experience could be observed between trials. All studies showed a significant difference in operative time, but while three studies found RIHR to take longer, one study reported shorter operative time with RIHR, altogether leading to a non-significant pooled estimate (MD 17.14; 95% CI −3.22 to 37.49). While one study showed significantly better ergonomics with RIHR, the other study reporting on that topic found ergonomics between the two approaches to be comparable. Surgeon task load varied substantially between the trials. The pooled estimate slightly favored RIHR but did not reach statistical significance (MD –4.50; 95% CI –16.78 to 7.78). Postoperative complication rate was not significantly different overall (OR 0.89, 95% CI 0.42 to 1.90), although one study reported significantly less complications after RIHR. RIHR shows comparable patient-centered outcomes to LIHR. At the moment, there is no evidence for significant benefits regarding task load and ergonomics.

**Supplementary Information:**

The online version contains supplementary material available at https://doi.org/10.1007/s11701-026-03805-8.

## Introduction

Inguinal hernia repair (IHR) is one of the most common surgical procedures with over 20 million operations performed worldwide per year [1]. While open repair remains the most frequently applied technique worldwide due to cost and limited availability of laparoscopy, guidelines recommend a minimally invasive approach if expertise is available due to faster recovery and lower rates of chronic pain [2, 3]. There is, however, an ongoing debate about whether robotic assistance in minimally invasive IHR is beneficial. Past studies have shown conflicting results regarding patient-centered outcomes such as pain and complications, but relatively consistently report increased operative time and higher cost for robotic approaches compared to conventional laparoscopic methods [4–8].

Because robotic surgery in general and the robotic approach in hernia repair in particular have been established rather recently, most studies were conducted during the surgeons’ learning curves, and thus their results must be interpreted carefully. A better predictor for the future adoption of robotic IHR than operative time during the learning curve might be surgeon-centered outcomes, such as ergonomics and surgeon task load, which have not been the focus of past meta-analyses [4–8]. Therefore, this systematic review and meta-analysis of randomized controlled trials comparing laparoscopic IHR (LIHR) and robotic IHR (RIHR) will review the current high-quality data landscape regarding surgeon-centered outcomes in the context of established patient-centered outcome parameters.

## Methods

This systematic review and meta-analysis was conducted and reported in accordance with the PRISMA 2020 guidelines [9]. The trial was not previously registered.

### Search strategy and study selection

A comprehensive literature search of the platforms Embase, the Cochrane Library, PubMed, and Web of Science was conducted on February 20, 2026. The aim was to identify randomized controlled trials comparing LIHR and RIHR in adult patients that reported at least one of the pre-specified endpoints (operative time, cost, surgeon task load, complications, postoperative pain, length of stay and readmissions). Non-comparative studies, retrospective analyses, case reports, abstracts, and reviews were excluded.

### Screening and data extraction

Literature screening was independently carried out by three authors (AR, FA, JS) and each entry was screened by at least two of them. Disagreements were resolved through discussion. The following data were extracted from the selected studies: first author, year of publication, study design, inclusion criteria, performed procedures, surgeon proficiency, number of participants, baseline characteristics (age, BMI, gender, ASA status), operative time, percentage of bilateral procedures, postoperative complications, surgeon task load measured by NASA-TLX score, surgeon ergonomics, postoperative pain, readmissions, length of hospital stay/number of unplanned inpatient stays, hernia recurrences and procedural costs. To ensure completeness, data were extracted from full texts, tables, figures, and supplementary materials.

### Risk of bias assessment and GRADE evaluation

A risk of bias assessment was conducted independently by two authors (AR, FA) using the Revised Cochrane risk-of-bias tool for randomized trials (RoB 2) [10]. Any disagreements were resolved through discussion or consultation with a third author.

The certainty of evidence for each outcome was assessed using the GRADE approach with GRADEpro GDT software [11]. Evidence from the included RCTs was initially rated as high certainty and downgraded based on risk of bias, inconsistency, indirectness, imprecision, and publication bias.

### Outcomes

Operative time, surgeon task load, ergonomics, postoperative complications, postoperative pain, and costs were defined as primary endpoints. Length of stay/unplanned inpatient stays, readmissions, and hernia recurrences were secondary endpoints. To improve comparability, outcomes of unilateral or simple procedures were used whenever possible.

#### Primary outcomes

##### Operative time

Measured in minutes based on each study's definition. If multiple durations were reported, the value closest to the "skin-to-skin" duration (first incision to skin closure) was utilized.

##### Surgeon task load

Recorded using NASA-TLX total scores [12, 13]. Where possible, RAW values were utilized, and subcategory scores were recorded.

The NASA-TLX is a rating scale to categorize the workload during given tasks [12]. It consists of the six subcategories mental demand, physical demand, temporal demand, performance, effort and frustration level. All single scores are then combined to a total score. The NASA-TLX score has been shown to correlate significantly with the perceived difficulty of a surgical procedure [13].

##### Ergonomics

All scores related to ergonomics, namely Rapid upper limb score (RULA) and Rapid entire body assessment (REBA) were recorded [14, 15].

The RULA is a tool focusing on the upper body to evaluate ergonomics by observing and scoring body posture during a given task. Additional points are given for the type of muscle use (static/repetitive) and added load. Higher scores generally indicate less favorable ergonomic circumstances [14]. The REBA follows a similar principle while giving a more detailed analysis of the lower limbs [15].

##### Postoperative complications

The number of participants who sustained a complication was compared. If available, the grade of the complication according to the Clavien-Dindo classification was noted [16].

##### Postoperative pain

All reported pain-related outcomes, along with the time of the survey, were recorded.

##### Cost

Procedural cost, defined as material and operating room cost, were measured in USD.

#### Secondary outcomes

##### Length of stay/unplanned admissions/readmissions

For inpatient procedures, the length of hospitalization was gathered; for ambulatory operations, the number of unanticipated admissions was recorded. If reported, readmission within the given timeframe were noted.

##### Hernia recurrence

The event rate of hernia recurrences during follow-up surveys was documented.

### Statistical analysis

All statistical analyses were conducted using R Version 4.5.2 [17]. For continuous variables, the mean and standard deviation (SD) were used. Data reported as medians and interquartile ranges (IQR) were first converted to means and SDs using the method described by Wan et al. [18]. Categorical variables were analyzed using odds ratios (OR) and 95% confidence intervals (CI). To evaluate study heterogeneity, Q-statistic, I^2^-statistic, and tau-statistic were employed. Pooled estimates were calculated using random-effects models. Sensitivity analyses were conducted by individually excluding studies to determine their impact on the pooled result; due to the small number of studies, no systematic exclusion was performed.

## Results

### Study selection

**Fig. 1:**
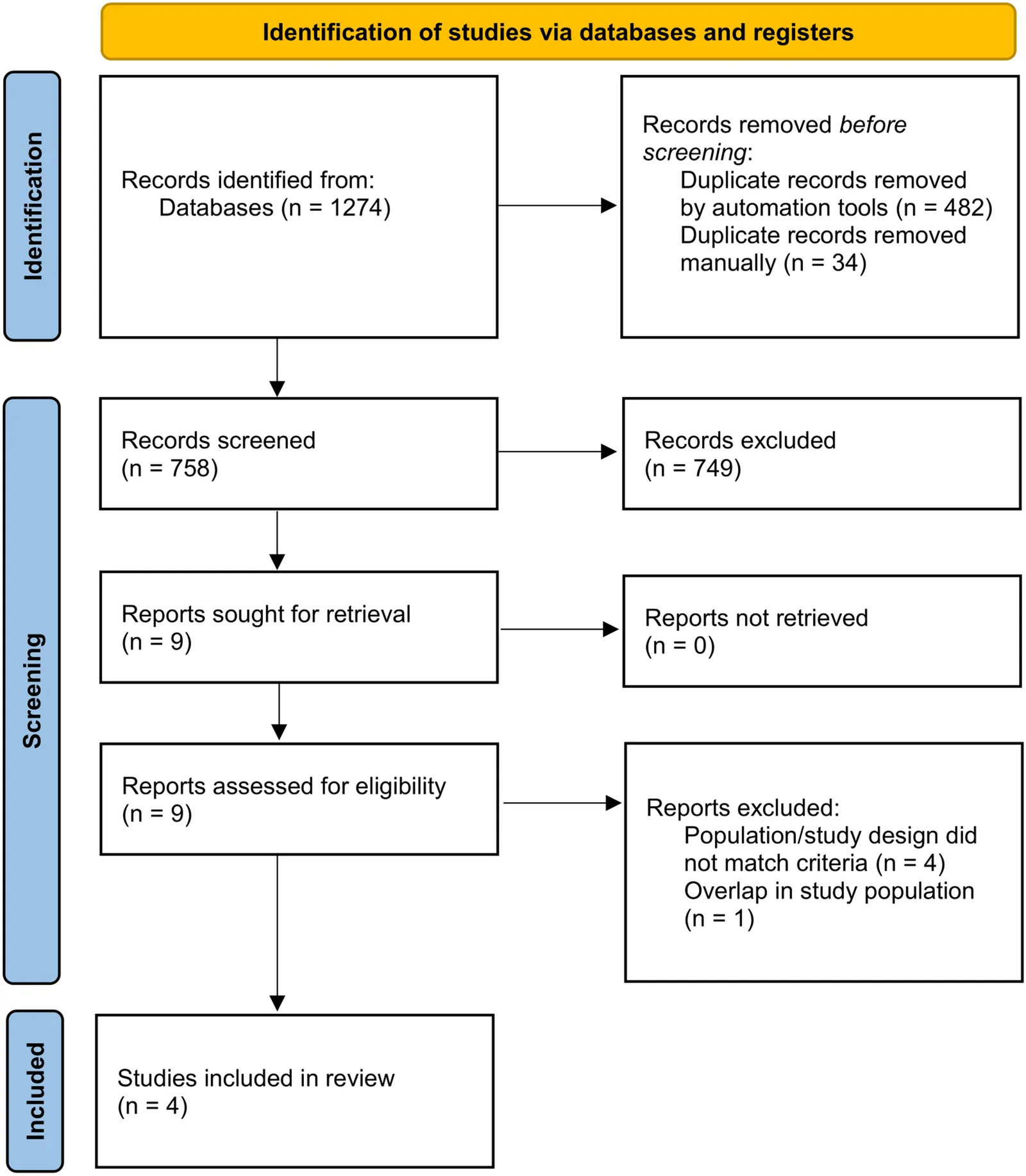
PRISMA flow diagram of included studies

A total of 1274 articles were identified through the systematic literature search. 516 were removed as duplicates. After screening titles and abstracts, 749 articles were excluded for not meeting the inclusion criteria of this systematic review and meta-analysis. Full-text screening was conducted for the remaining 9 articles, after which 4 articles were excluded (Fig. 1).

A total of five studies were identified [19–23]. One of those (DIRECT) was excluded due to an overlapping study population with the ROLAIS trial. Because operative time was better specified in the DIRECT trial, this information was retrieved from there, even though the study itself was not included.

For the RIVAL trial, a 1- and 2-year follow-up was published by Miller et al. [24]. Long-term outcomes reported exclusively in this secondary paper are included in this meta-analysis to enhance its informative value and are clearly labeled.

### Risk of bias assessment and GRADE evaluation

The risk of bias assessment revealed low concern for the studies by Angehrn et al. and Valorenzos et al. as well as some concerns for bias for the studies by Prabhu et al. and Dixon et al. Results for the GRADE Evaluation are reported with each outcome and presented in a Summary of Findings Table which can be found in the Supplement.

### Baseline characteristics

A total of 482 patients were included. 252 underwent robotic IHR, which were all performed as transabdominal preperitoneal hernioplasty (TAPP), and 230 underwent conventional minimal invasive IHR, either laparoscopic TAPP or totally extraperitoneal hernioplasty (TEP).

In summary, the baseline characteristics were comparable: Mean age was 57.84 years (standard deviation (SD) 14.85) in the RIHR-group and 59.65 years (SD 14.17) in the LIHR-group (p = 0.172). 90.9% of patients in the RIHR group and 88.7% in the LIHR group were male (p = 0.428). The pooled mean body mass index (BMI) was 25.43 kg/m^2^ (SD 3.51) for patients undergoing RIHR and 25.56 kg/m^2^ (SD 3.59) in LIHR (p = 0.701). Three studies reported physical status according to the American Society of Anesthesiologists (ASA status) as surrogate for comorbidity. More than 80% in both groups had an assigned ASA status of I or II and there was only one reported ASA IV patient in the laparoscopic group.

The required surgeon qualification differed greatly between the studies. Prabhu et al. demanded ≥ 25 each robotic and laparoscopic procedures, Dixon et al. required at least 20 cases using the respective modality and Angehrn et al. mandated at least 30 conducted procedures of each in the past year as well as > 50 advanced robotic procedures in general, whereas the participating surgeons in the ROLAIS trial were reported to have performed at least 100 R-TAPPs and multiple hundred L-TAPPs prior to the study.

### Primary outcomes

#### Operative time

Angehrn et al. Prabhu et al. and Dixon et al. reported significantly longer operative times with robotic assistance, whereas Valorenzos et al. showed a significant reduction in operative time compared to the laparoscopic approach. Consequently, the pooled estimate favors LIHR, but fails to reach statistical significance (Mean Difference (MD) 17.14; 95% CI − 3.22 to 37.49) due to very high heterogeneity (I^2^ = 98.7%).

**Fig. 2:**
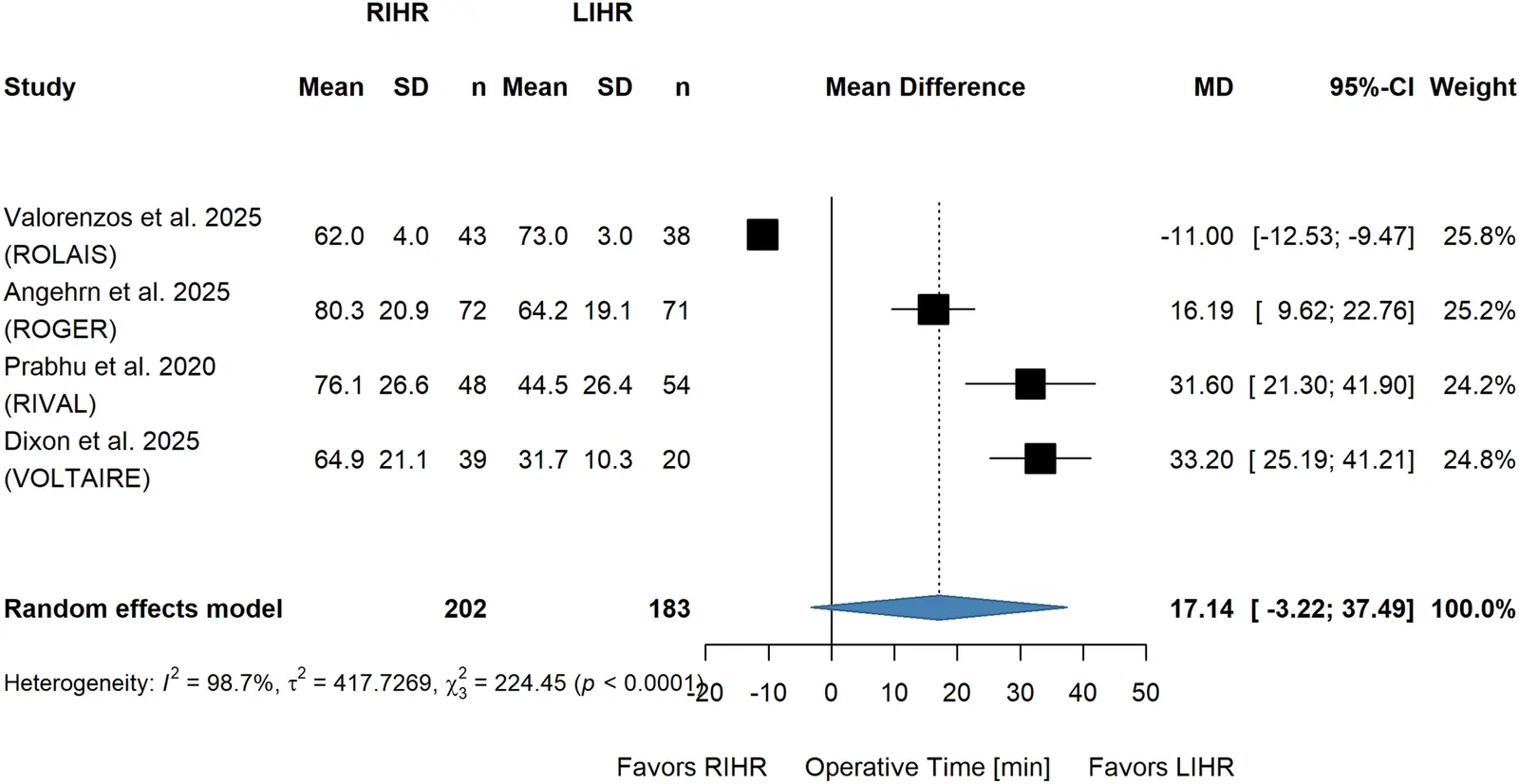
Forest plot for operative time

Following a sensitivity analysis excluding Valorenzos et al., the mean difference shifted to 24.71 min and reached statistical significance (95% CI 20.15 to 29.27), confirming this study's role as an outlier. The certainty of evidence was rated as high (Fig. 2).

#### Surgeon task load

**Fig. 3:**
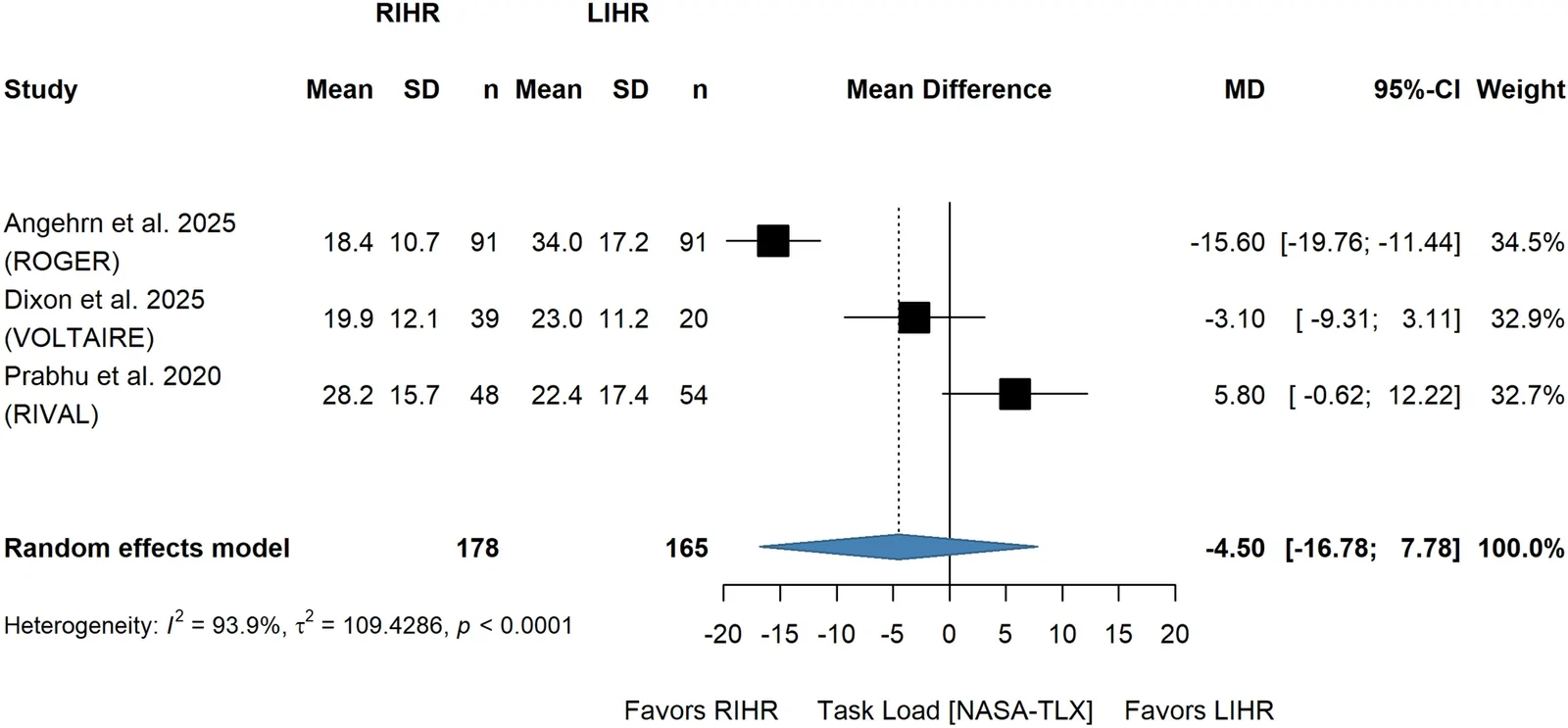
Forest plot for surgeon task load

Three of the studies measured surgeon task load according to NASA-TLX. Angehrn et al. demonstrated a significantly lower task load during RIHR (MD − 15.60; 95% CI − 19.76 to − 11.44). Dixon et al. also reported results favoring the robotic approach, though they were not statistically significant (MD − 3.10; 95% CI − 9.31 to 3.11), while Prabhu et al. found a non-significant advantage for the laparoscopic method (MD 5.80, 95% CI − 0.62 to 12.22). In summary, RIHR is slightly favored overall (MD − 4.50; 95% CI − 16.78 to 7.78), but due to high heterogeneity among the three studies (I^2^ = 93.9%) statistical significance was not reached and certainty of evidence was rated as low (Fig. 3).

#### Ergonomics

Two studies reported ergonomic measurements. The study of Prabhu et al. used the RULA score and while showing a significantly worse ergonomic outcome concerning the left upper limb score for RIHR (5.6 vs. 4.92, p = 0.01), it did not report a significant difference in the grand composite score (10.3 vs. 9.80 on the left side, p = 0.31, and 10.2 vs. 10.1 on the right side, p = 0.94). Ergonomics were measured using the REBA score in the study of Dixon et al., and here a significant difference in favor of RIHR was found (median 3 (2) vs. 5 (1), p < 0.001).

#### Postoperative complications

**Fig. 4:**
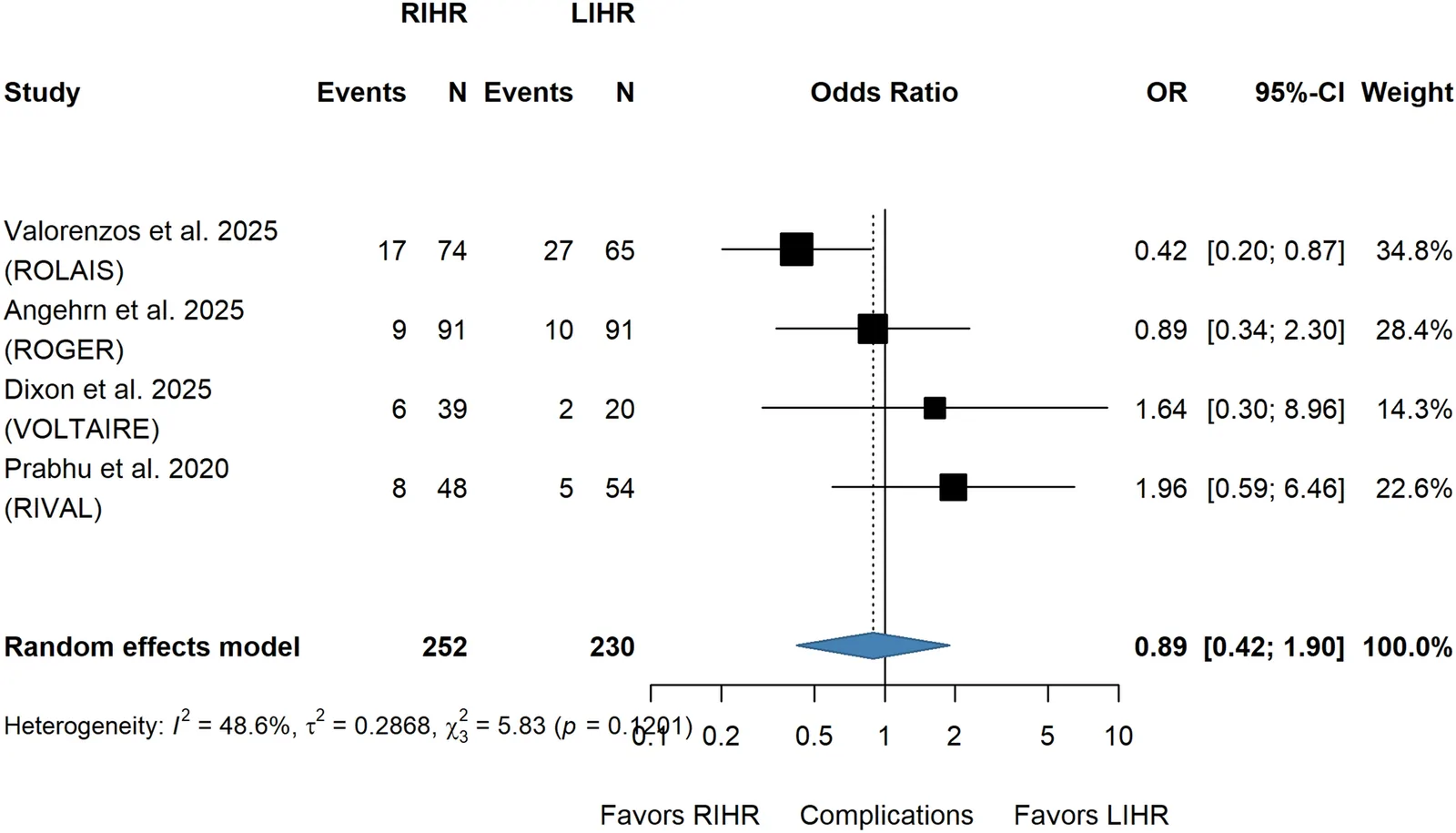
Forest plot for complications

A total of 84 postoperative complications were reported in all of the four studies: 40 in the RIHR-group and 44 in the LIHR group. Valorenzos et al. showed a significantly higher complication rate in the LIHR-group, whereas the differences in the other studies were not statistically significant. Overall, the pooled OR was 0.89, slightly favoring the robotic approach, but lacking statistical significance (95% CI 0.42 to 1.90) (Fig. 4). Notably, 93% of the reported complications were classified as Clavien-Dindo grade I or II.

A more detailed list is available in the supplementary material. Due to imprecision the certainty of evidence was downgraded to moderate certainty.

#### Postoperative pain

Because the studies varied widely in how and when they measured postoperative pain, a pooled meta-analysis could not be conducted. Both Prabhu et al. and Angehrn et al. showed a similar evolution of pain during the first 30 postoperative days. Dixon et al. only reported pain at 14 days postoperatively, noting no significant difference. Valorenzos et al. measured chronic postoperative inguinal pain and reported almost twice as many affected patients in the LIHR group compared to the RIHR group, though this difference was not statistically significant (12.3% vs. 6.8%; OR 1.94; 95% CI 0.61 to 6.72). Overall, high certainty of evidence was reached. Detailed information can be seen in the supplement.

#### Cost

**Fig. 5:**
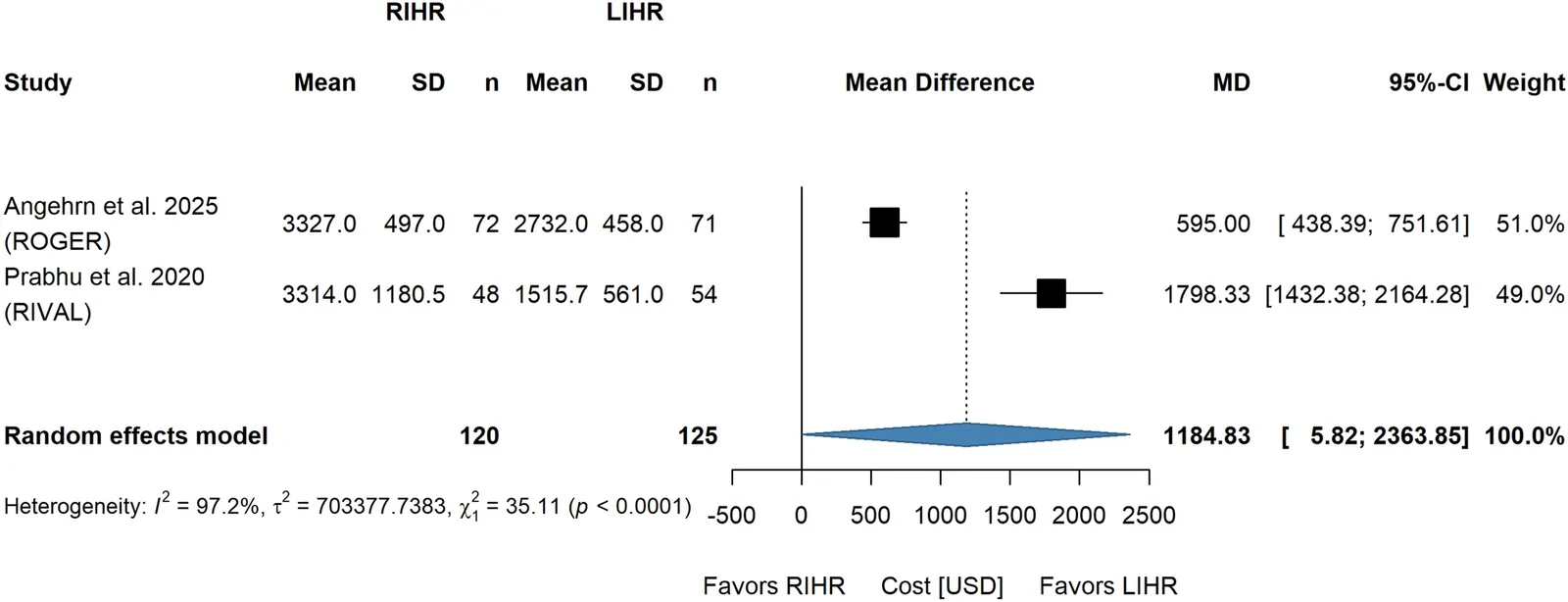
Forest plot for cost

Both Angehrn et al. and Prabhu et al. reported significantly higher expenses for the robotic approach in unilateral IHR, resulting in a significant pooled estimate (MD 1184.83, 95% CI 5.82 to 2363.85). The certainty of evidence was high (Fig. 5).

### Secondary outcomes

#### Length of stay / unplanned admissions/readmissions

Angehrn et al. and Dixon et al. reported no significant differences in the length of hospital stay between groups. In the study by Prabhu et al. where all operations were planned as outpatient procedures, unanticipated admissions were necessary for two patients in the LIHR-group (3.7%) and four in the RIHR-group (8.3%; p = 0.420). Similar parameters applied to Valorenzos et al., though in their study, significantly more patients required unplanned inpatient care following LIHR (18.5% vs. 4.1%; OR 5.36; 95% CI 1.61 to 24.38). Additionally, in this study significantly more patients in the LIHR group were readmitted within 6 months (12.3% vs. 1.4%; OR 10.25; 95% CI 1.81 to 192.86). Readmissions were also reported by Dixon et al. and within 30 days only one readmission was registered; this patient had a RIHR (2.6% vs. 0.0%; p = 0.47). Due to high risk of bias only moderate certainty of evidence could be achieved.

#### Hernia recurrence

Only two studies initially had follow-up periods long enough to evaluate recurrences. Angehrn et al. reported one recurrence in the laparoscopic group at 12 months, while Valorenzos et al. discovered one recurrence in the robotic group at 6 months.

In the Follow-Up of the RIVAL trial, one recurrence was observed in each group after two years. Certainty of evidence was high.

## Discussion

Although previous meta-analyses on the comparison of RIHR and LIHR exist, up until now the focus has been patient-centered and economic outcomes and not surgeon-centered outcomes like task load and ergonomics [4–7].

### Primary outcomes

#### Operative time

Operative time has been a much-discussed issue in previous analyses [4–8].

In accordance to already published data, this meta-analysis found a higher operation time for RIHR, although the individual studies showed heterogeneous results. Operation time was analyzed only for unilateral/simple procedures to improve comparability between the studies. There is an exception for the study of Dixon et al., where data was not available for those subgroups, but as both groups had only 8% and 10% bilateral hernias, their effect on the total outcome is likely similar in both groups and not predominant. None of the studies reported a mean docking time of more than ten minutes, regardless of the used robotic platform. As the mean difference in operation time in this meta-analysis is 17.14 min summarizing all four studies and 24.71 min when excluding Valorenzos et al. for sensitivity analysis, docking time alone cannot explain the observed prolongation.

The only study showing shorter operation time for the robotic approach was Valorenzos et al. which at the same time had by far the highest proficiency requirements for participating surgeons. The other three studies may have been conducted prior to the completion of the surgeons’ learning curves. Since RIHR is the newer modality, it can be assumed participating surgeons in these trials were generally less experienced with the robotic platform compared to their laparoscopic backgrounds. This has already been suggested in previous analyses and can be stressed further through the addition of a fourth RCT (ROGER) [6]. There is evidence showing a decrease in operative time during progression on the learning curve of the robotic approach [25–27]. Furthermore, there are indications that 20–30 procedures, which is the number which all of the here discussed studies, apart from Valorenzos et al., required participating surgeons to have performed previous to participation in their study, might not yet be the end of the learning curve and over 100 procedures for RIHR, as required in the study of Valorenzos et al., could represent a more reliable definition of mastery of RIHR [28]. Those studies are, however, only indicators and a definitive statement regarding future development cannot be made yet. Now, evidence suggests that operative time in RIHR is longer than in LIHR. Further high-value research is needed to determine whether operative time can be significantly reduced with progression on the learning curve.

Only Angehrn et al. reported explicitly on bilateral (primary) hernias and Valorenzos et al. provided an operation time analysis for complex (bilateral and/or large and/or recurrent) hernias. As group composition and inclusion criteria were too heterogeneous, a statistical analysis whether RIHR is more beneficial in patients with bilateral or recurrent hernias or history of other previous lower abdominal surgery could not be conducted. There are indicators that RIHR is more efficient in bilateral hernias [4]. However, to the authors’ knowledge, evidence regarding the immediate comparison of the efficiency of the robotic approach compared to conventional laparoscopic IHR in complex cases (recurrent hernia, previous lower abdominal surgery) is limited and should be addressed in further research.

#### Surgeon task load

The results concerning surgeon task load were mixed. The only significant difference was found by Angehrn et al., which was in favor of the robotic approach. Looking at the subcategories, only the mental workload was reported to have been higher in RIHR, which might be due to not having completed the learning curve yet. Higher mental demand was also reported by Prabhu et al. The study of Angehrn et al. reports lower demand regarding physical workload and frustration favoring RIHR, a result which is not reciprocated by the other studies. Prabhu et al. even registered higher frustration with RIHR. One possible explanation might be the differing laparoscopic approaches. Angehrn et al. was the only study to perform total extraperitoneal hernioplasty (TEP) while all others used the transabdominal preperitoneal approach (TAPP). As already discussed in the ROGER study, the TEP seems to yield a subgroup of surgeries which are performed relatively easily and one which requires a much higher effort, while no similar compartmentalization was observed in the robotic TAPPs in this study. It is possible that the subgroup of difficult TEPs drove up the score for frustration in the LIHR group, which could explain why similar results were not observed in the other studies. Unfortunately, surgeon task load is not measured in the study of Valorenzos et al., which had the highest surgeon proficiency, to show whether mental demand and frustration decrease in total and especially in RIHR with increasing experience.

As discussed regarding operation time, there are indications towards a more beneficial role of the robotic approach in bilateral hernias as opposed to simple unilateral hernias [4]. Surgeon task load was not explored in this context or in the comparison of simple to complex procedures so far. As a specific breakdown of surgeon task load into unilateral/bilateral or simple/complex procedures is not available in the included studies of this meta-analysis, no definitive statement can be made.

Current literature suggests to use robotic surgery mainly in anatomical complex procedures instead of all minimally invasive cases [29, 30]. Inguinal hernias are generally not considered to be part of the first group; however, there might be exceptions in very large, inguinoscrotal or recurrent hernias, which provide a more challenging anatomy. The same rationale applies to patients with history of extensive lower abdominal surgery, where tissue preparation can be more difficult due to adhesions. Further research is needed to determine whether the robotic approach is associated with relatively more beneficial outcomes in those cases regarding surgeon task load compared to the conventional laparoscopic method.

#### Ergonomics

While the study of Rival et al. could not find a significant difference regarding ergonomics between the two groups, the study of Dixon et al. found the robot to be beneficial. There are several possible reasons for this discrepancy. First, the study of Rival et al. was conducted five years prior to the study of Dixon et al., therefore overall experience with robotic surgery is likely to have been higher in the latter study. The better accommodation to robotic surgery in general might influence the ergonomic strain. Second, the study of Prabhu et al. used the da Vinci Xi/Si system (closed console system) while Dixon et al. used the Versius system (open console system). While evidence is still limited, there are indications pointing towards improved ergonomics with open console systems, which might explain the observed difference between the two studies [31, 32].

Overall, despite early assumptions, the pooled data of these trials failed to show a significant advantage for the robotic approach regarding either ergonomics or surgeon workload.

#### Postoperative complications

In summary, complication rates in all studies and for all groups were low, with less than 20% in total. Valorenzos et al. reported significantly fewer complications in the RIHR group, primarily due to lower rates of chronic postoperative inguinal pain (CPIP) and hematomas, which might be a consequence of better visualization and therefore tissue handling. It is possible that this results from higher surgeon proficiency with better use of the robotic advantages compared to the other studies, where results were not significant, but tended towards slightly more complications with RIHR. Beyond statistical significance, the clinical relevance of these findings must also be addressed. 93% of the complications could be classified as Clavien-Dindo grade I or II, not requiring invasive therapy like reintervention or reoperation. Still, especially the evolution of chronic pain is of great relevance for patients and could impair their quality of life [33]. Concerning this meta-analysis, Angehrn et al. and Prabhu et al. could not detect a difference in quality of life. Valorenzos et al. as the study with the highest complication rates and largest difference between groups did not report on this topic.

#### Postoperative pain

No significant differences were found regarding postoperative pain. The study of Valorenzos et al. reported a non-significantly higher rate of CPIP, which was not measured in the other studies. Angehrn et al. and Prabhu et al. measured pain at multiple occasions and still did not find significant differences. Altogether this suggests that RIHR and LIHR result in comparable postoperative pain and both methods are therefore viable.

#### Cost

In accordance with previous literature this meta-analysis showed higher costs for the robotic approach Prabhu et al. found RIHR to be more than twice as expensive as LIHR, while the difference was not as large in the study by Angehrn et al. [4, 5]. Looking at the detailed breakdown, the balloon and suitable trocar as well as the glue for mesh fixation account for almost all material costs in the LIHR group in the ROGER study. As this material is unique to the TEP approach, a similar difference as in the RIVAL trial would likely have been achieved if a TAPP had been performed. In summary, RIHR at the moment remains more expensive than LIHR. A huge driver of this difference is the prolonged operative time in RIHR [34]. As discussed above, a reduction of operative time is possible along the learning curve and might be accompanied by a reduction of cost. High-level evidence on this topic is yet to be produced.

### Secondary outcomes

#### Length of stay / unplanned admissions / readmissions

The only study finding significant differences regarding length of stay or admissions was the study of Valorenzos et al., where 18.5 vs. 4.1% of the patients in the LIHR group required inpatient care and 12.3 vs. 1.4% of the patients in the LIHR were readmitted within 6 months. The reasons were not further specified in the study but might be associated with the according higher complication rate in the LIHR group in this study. All other studies showed comparable results between the groups concerning this parameter. The GRADE Evaluation showed only moderate certainty of evidence because length of stay and admissions might be biased by knowledge of the intervention group, especially as in all studies except for the ROGER study investigators were not blinded.

#### Hernia recurrence

Recurrence rate was low overall (0.9%), which aligns with the current literature [35, 36]. The comparison of the LIHR and the RIHR group did not reveal significant differences. As the follow-up period was 6, 12 or 24 months in the respective studies, the absolute numbers might have been underestimated and long term follow up is needed to determine whether recurrence rates between RIHR and LIHR are different.

### Limitations

Several limitations must be acknowledged. First, the chosen laparoscopic technique varied, with Angehrn et al. utilizing TEP while the others employed TAPP. Although both methods yield comparable patient-centered outcomes [37, 38], their respective impacts on surgeon task load and ergonomics remain under-explored. From the authors’ point of view, the TEP procedure might be perceived as more straining, which could explain the significant advantage towards the robotic approach in the ROGER study. The comparison of TEP with R-TAPP in this study and the inclusion in this meta-analysis is still reasonable as TEP represents the default approach for inguinal hernia at their institution.

Second, the number of included studies and participants is relatively small. By strictly focusing on randomized controlled trials to ensure a high level of evidence, statistical power was limited, and only the cost analysis yielded a pooled statistically significant result. Similarly, the sample sizes of the included studies are relatively small. The resulting limited power of the trials might mask significant differences. At the same time, comparison of the results between the studies becomes more difficult due to the high heterogeneity.

Nevertheless, as the first meta-analysis to focus specifically on surgeon workload and ergonomics in this context, it provides a valuable summary of current evidence and highlights critical avenues for future research.

### Considerations regarding the application of RIHR

Considering that current data shows no clear benefit for RIHR regarding patient- or surgeon-centered outcomes, but longer operative times and higher cost, a critical evaluation of routine RIHR is necessary. There are, however, specific situations in which the use of RIHR might be justified.

First, it can serve as a safe training procedure for surgeons new to robotic platforms.

Second, RIHR is associated with fewer peritoneal tears and lower conversion rates [4, 39, 40]. This suggests that the three-dimensional visualization and wristed instrumentation might translate into a more controlled and precise dissection. There are two potential implications to this theory, which require further research: On one hand, more precise dissection could improve identification and preservation of the inguinal nerves and thereby reduce the rate of CPIP occasionally seen after extended lateral dissection in laparoscopic repair. On the other hand, this same precision might facilitate dissection and suturing during primary closure of large defects (EHS M3), the use of which is debated analogous to fascial closure in ventral hernia repair, once again while sparing the nerves in this region.

Further prospective studies with large sample sizes are required to determine whether these theoretical considerations translate into clinically relevant results.

### Summary

In summary, there are no significant differences in complications and postoperative pain between robotic and laparoscopic inguinal hernia repairs. Operative times and associated costs currently remain higher for the robotic approach, though this disparity may narrow with increased surgical experience and reduced fixed equipment costs. Superiority of the robotic approach regarding surgeon task load and ergonomics cannot be proven based on this study. Further large-scale randomized controlled trials with sufficient sample sizes and high surgeon proficiency are required. Based on the data of this study, a recommendation to apply robotic surgery in standard IHR cannot be given. To determine whether robotic surgery should be applied in IHR, future research should specifically evaluate the impact of the learning curve on surgeon task load and cost-effectiveness and investigate whether the robotic approach yields distinct advantages in anatomically complex or recurrent hernias.

## Conclusion

In this meta-analysis of randomized controlled trials, there were no significant differences in surgeon workload, ergonomics, or clinical outcomes between robotic and laparoscopic inguinal hernia repairs. However, robotic IHR was associated with longer operative times and higher costs. These findings question the value of robotic surgery for routine inguinal hernias. While RIHR may serve as a useful training case for novice robotic surgeons or theoretically aid in complex clinical scenarios, further studies are needed to identify specific subpopulations that might truly justify the robotic approach.

## Supplementary Information

Below is the link to the electronic supplementary material.

Supplementary file1 (DOCX 774 KB)

## Data Availability

The datasets generated and analyzed during the current study are available from the corresponding author on reasonable request.
