# Supplementary material for: Surgeon task load, ergonomics, and clinical outcomes in laparoscopic versus robotic-assisted inguinal hernia repair: a systematic review and meta-analysis of randomized controlled trials
Source: J Robot Surg. 2026 Oct 7;20(1):1070. doi: 10.1007/s11701-026-03805-8 (PMC13645844; doi:10.1007/s11701-026-03805-8)
Supplement: Supplementary file 1 [file 11701_2026_3805_MOESM1_ESM.docx]

**Journal of Robotic Surgery**

**Comparative Outcomes of Laparoscopic and Robotic-Assisted Inguinal Hernia Repair: A Systematic Review and Meta-analysis of Randomized Controlled Trials**

Anna Rümmele-Vogt*^+1^, Julian Süsstrunk^+1^, Marie-Thérèse Böttcher^1^, Johannes Baur^1^, Beat P Müller^1^, Daniel C Steinemann^1++^, Fiorenzo V. Angehrn^1++^

^1^Clarunis, Department of Visceral Surgery, University Digestive Health Care Centre, St. Clara Hospital and University Hospital Basel, Basel, Switzerland.

*Corresponding Author

+Shared first authorship

++Shared last authorship

**Fig. S1** Search Terms

PubMed
("hernia, inguinal"[MeSH Terms] OR “inguinal hernia”[MeSH Terms] OR "inguinal hernias"[MeSH Terms] OR "inguinal hernia"[Title/Abstract] OR "groin hernia"[Title/Abstract])
AND
 ("Robotics"[MeSH Terms] OR "Robotic Surgical Procedures"[MeSH Terms] OR "procedure, robotic surgical"[MeSH Terms] OR "surgical procedure, robotic"[MeSH Terms] OR "robot*"[Title/Abstract] OR "robot-assisted"[Title/Abstract] OR "robot-assisted"[Title/Abstract])
AND
("Laparoscopy"[MeSH Terms] OR "procedure, laparoscopic surgical"[MeSH Terms] OR "surgical procedure, laparoscopic"[MeSH Terms] OR "laparoscop*"[Title/Abstract] OR "minimally invasive"[Title/Abstract])
🡪 453 results

Embase
 ('inguinal hernia'/exp OR 'inguinal hernia') AND ('robot-assisted procedure'/exp OR 'robot-assisted procedure') AND ('laparoscopic surgery'/exp OR 'laparoscopic surgery' OR 'minimally invasive procedure'/exp OR 'minimally invasive procedure')
🡪 336 results

Web of Science
('inguinal hernia'/exp OR 'inguinal hernia') AND ('robot-assisted procedure'/exp OR 'robot-assisted procedure') AND ('laparoscopic surgery'/exp OR 'laparoscopic surgery')
🡪 445 results

Cochrane
"inguinal hernia" in All Text AND "laparoscop*" OR "minimally invasive surgery" OR "minimally invasive surgeries" OR "minimally-invasive surgery" OR "minimally-invasive surgeries" OR "laparoscopic" in Title Abstract Keyword AND "robotic" OR "robotically" OR "robot-assisted" OR "robotic-assisted" OR "robot assisted" OR "robotic assisted" OR "robot" in Title Abstract Keyword
🡪 40 results

**Figure S2** PRISMA 2020 Checklist

**Figure S3** PRISMA 2020 for Abstracts Checklist

**Table S1** Characteristics of the included studies

| **Study** | **First author** | **Year** | **Design** | **Population / eligibility** | **Intervention** | **Control** | **Robotic platform** | **Surgeon proficiency** | **Centre(s)** |
| --- | --- | --- | --- | --- | --- | --- | --- | --- | --- |
| **ROGER** | Angehrn | 2025 | RCT | Adults (≥18 y), elective, uni-/bilateral; no recurrent hernias | R-TAPP | L-TEP | da Vinci Xi | ≥30 respective procedures in the past year; >50 advanced robotic procedures overall | Single centre |
| **RIVAL** | Prabhu | 2020 | RCT | Adults (≥21 y), elective, unilateral; recurrences included | R-TAPP | L-TAPP | da Vinci Xi / Si | Attendings; ≥25 robotic and ≥25 laparoscopic procedures (not specified whether hernia-specific) | 6 centres |
| **ROLAIS** | Valorenzos | 2025 | RCT | Adults (≥18 y), elective, uni-/bilateral; recurrences included | R-TAPP | L-TAPP | da Vinci Xi | Formal robotic certification; ≥100 R-TAPPs and hundreds of L-TAPPs | Single centre |
| **VOLTAIRE** | Dixon | 2025 | RCT | Adults (≥18 y), elective, uni-/bilateral; only recurrences after previous open repair | R-TAPP | L-TAPP | Versius | ≥20 cases with the relevant modality | Single centre |

**Table S2** Number of participants

| **Study** | **First author** | **Number of participants, n** | | |
| --- | --- | --- | --- | --- |
|  |  | **Total** | **Intervention** | **Control** |
| **ROGER** | Angehrn | 182 | 91 (72 unilateral) | 91 (71 unilateral) |
| **RIVAL** | Prabhu | 102 | 48 | 54 |
| **ROLAIS** | Valorenzos | 139 | 74 (43 simple, 58 unilateral) | 65 (38 simple, 57 unilateral) |
| **VOLTAIRE** | Dixon | 59 | 39 (36 unilateral) | 20 (18 unilateral) |

.

**Table S3** Operative and early postoperative outcomes

| **Study** | **First author** | **Operation time (min)** | | | **NASA-TLX** | | | **Cost** | | **Complications, n** | | |
| --- | --- | --- | --- | --- | --- | --- | --- | --- | --- | --- | --- | --- |
|  |  | **Definition** | **Intervention** | **Control** | **Instrument** | **Intervention** | **Control** | **Intervention** | **Control** | **Intervention** | **Control** | **Comment on complications** |
| **ROGER** | Angehrn | Incision to suture | Unilateral 80.34 (20.93); bilateral 126.61 (19.78) | Unilateral 64.15 (19.15); bilateral 102.65 (23.76) | RAW | 18.4 (10.7) | 34 (17.2) | 3327 (497) | 2732 (458) | 9 | 10 | One Clavien–Dindo grade IIIb in the laparoscopic group; otherwise grade I/II |
| **RIVAL** | Prabhu | Skin incision to closure | 75.5 [59.0–93.8] | 40.5 [29.2–63.8] | RAW and overall | RAW 28.2 (15.7); overall 27.5 (15.6) | RAW 22.4 (17.4); overall 21.9 (18.2) | 3258 [2568–4118] | 1421 [1196–1930] | 8 | 5 | No Clavien–Dindo classification reported; from the descriptions, one grade IIIa in the laparoscopic group, otherwise grade I/II |
| **ROLAIS** | Valorenzos | see DIRECT | see DIRECT | see DIRECT | — | — | — | — | — | 17 | 27 | Two Clavien–Dindo grade IIIb in the laparoscopic group; one IIIb and one IVa in the robotic group; otherwise grade I/II |
| **VOLTAIRE** | Dixon | Not further specified | 64.9 (21.1) | 31.7 (10.3) | Modified | 19.9 (12.1) | 23 (11.2) | — | — | 6 | 2 | Only grade I and II |
| **DIRECT** | Arunthavanathan | Veress-needle placement to skin closure | Simple 62 ± 4; complex 83 ± 4 | Simple 73 ± 3; complex 105 ± 7 | — | — | — | — | — | — | — | Companion report providing operation times for the ROLAIS cohort |

**Table S4** Postoperative outcomes and patient-reported measures

| **Study** | **First author** | **Length of stay / admissions** | | **Readmissions** | | **Recurrences** | | **Ergonomics** | | **Pain** | |
| --- | --- | --- | --- | --- | --- | --- | --- | --- | --- | --- | --- |
|  |  | **Intervention** | **Control** | **Intervention** | **Control** | **Intervention** | **Control** | **Intervention** | **Control** | **Intervention** | **Control** |
| **ROGER** | Angehrn | Unilateral usually planned ambulatory; 12 inpatients 1.08 (0.28); bilateral 1.33 (0.59) | Unilateral usually planned ambulatory; 9 inpatients 1.75 (1.39); bilateral 1.30 (0.66) | — | — | None at 6 and 12 months | None at 6 months; one at 12 months | — | — | NRS at rest, median (IQR): 24 h 2.00 (1.00–3.00); 7 d 1.00 (0.00–1.00); 30 d 0.00 (0.00–1.00); 6 mo 0.00 (0.00–0.00); 12 mo 0.00 (0.00–0.00).  NRS on coughing, median (IQR): 24 h 4.00 (2.00–7.00); 7 d 3.00 (1.00–4.00); 30 d 0.00 (0.00–1.00); 6 mo 0.00 (0.00–0.00); 12 mo 0.00 (0.00–0.00). | NRS at rest, median (IQR): 24 h 2.00 (1.00–3.00); 7 d 0.00 (0.00–1.00); 30 d 0.00 (0.00–0.00); 6 mo 0.00 (0.00–0.00); 12 mo 0.00 (0.00–0.00).  NRS on coughing, median (IQR): 24 h 5.00 (2.00–7.00); 7 d 2.00 (1.00–3.00); 30 d 0.00 (0.00–0.00); 6 mo 0.00 (0.00–0.00); 12 mo 0.00 (0.00–0.00). |
| **RIVAL** | Prabhu | 5.75 h [5.00–7.00]; 4 unanticipated admissions | 5.11 h [4.00–7.00]; 2 unanticipated admissions | — | — | 1 at 2 years (Miller et al. follow-up) | 1 at 2 years (Miller et al. follow-up) | Grand composite score 10.3 (left), 10.2 (right); left upper-limb score significantly worse (5.62 vs 4.93) | Grand composite score 9.80 (left), 10.1 (right) | Baseline 15.2; +5.53 at 1 week; –7.00 at 1 month | Baseline 18.8; +4.60 at 1 week; –7.92 at 1 month |
| **ROLAIS** | Valorenzos | Planned ambulatory; 3 treated as inpatients | Planned ambulatory; 12 treated as inpatients | 1 within 6 months | 8 within 6 months | 1 at 6 months | None at 6 months | — | — | Chronic postoperative inguinal pain: 5 | Chronic postoperative inguinal pain: 8 |
| **VOLTAIRE** | Dixon | 0 [1] | 0 [1] | 1 within 30 days | None within 30 days | — | — | REBA 3 [2] | REBA 5 [1] | Pain score at 14 days 0 [2] | Pain score at 14 days 1 [2] |

**Table S5** GRADE Evaluation Summary of Findings
